# Japanese studies on neural circuits and behavior of *Caenorhabditis elegans*

**DOI:** 10.3389/fncir.2013.00187

**Published:** 2013-11-28

**Authors:** Hiroyuki Sasakura, Yuki Tsukada, Shin Takagi, Ikue Mori

**Affiliations:** ^1^Laboratory of Molecular Neurobiology, Division of Biological Science, Nagoya UniversityNagoya, Japan; ^2^Laboratory of Brain Function and Structure, Division of Biological Science, Nagoya UniversityNagoya, Japan

**Keywords:** *C. elegans*, neural circuits, learning and memory, plastic behavior, behavioral genetics, studies on Japan

## Abstract

The nematode *Caenorhabditis elegans* is an ideal organism for studying neural plasticity and animal behaviors. A total of 302 neurons of a *C. elegans* hermaphrodite have been classified into 118 neuronal groups. This simple neural circuit provides a solid basis for understanding the mechanisms of the brains of higher animals, including humans. Recent studies that employ modern imaging and manipulation techniques enable researchers to study the dynamic properties of nervous systems with great precision. Behavioral and molecular genetic analyses of this tiny animal have contributed greatly to the advancement of neural circuit research. Here, we will review the recent studies on the neural circuits of *C. elegans* that have been conducted in Japan. Several laboratories have established unique and clever methods to study the underlying neuronal substrates of behavioral regulation in *C. elegans*. The technological advances applied to studies of *C. elegans* have allowed new approaches for the studies of complex neural systems. Through reviewing the studies on the neuronal circuits of *C. elegans* in Japan, we will analyze and discuss the directions of neural circuit studies.

## THE NERVOUS SYSTEM OF *C. elegans*

Understanding the mechanisms underlying neural operation and processing is a major goal in neuroscience. One approach is to study a simple neural circuit, in which neurons and neural pathways can be identified and diagrammed for analysis. Another approach is the application of molecular genetic techniques to reveal the molecular components that govern the neural functions and clarify the site of action of molecules in brain (Brenner, 1974; Quinn et al., 1974).

The soil nematode *Caenorhabditis elegans* is an ideal organism for studying neural circuits and related behavior, since it completely fulfills the above-mentioned criteria. The nervous system of *C. elegans* consists of only 302 neurons and the entire neuronal system of *C. elegans* is composed of approximately 5,000 synapses and 600 gap junctions, as revealed by electron microscopic analysis (Brenner, 1974; Ward et al., 1975; Ware et al., 1975; Sulston et al., 1983; White et al., 1986). Major neurotransmitters in mammals such as glutamate, acetylcholine, GABA, and monoamines all occur in the nervous system of *C. elegans* (Jorgensen, 2005; Brockie and Maricq, 2006; Chase and Koelle, 2007; Rand, 2007; Li and Kim, 2008). In addition, homologs of genes important for neural development and function in mammals are found in the *C. elegans* genome, suggesting that findings on the *C. elegans* nervous system will be useful for understanding the human brain (Bargmann, 1998; C. elegans Sequencing Consortium, 1998; Hobert, 2005). Furthermore, calcium imaging and optogenetics have been effectively applied to the *C. elegans* nervous system, yielding valuable information about dynamics of neurons and circuit in living animals (Schafer, 2005, 2006; Kerr, 2006; Han and Boyden, 2007; Zhang et al., 2007; Tian et al., 2009; Xu and Kim, 2011).

**FIGURE 1:**
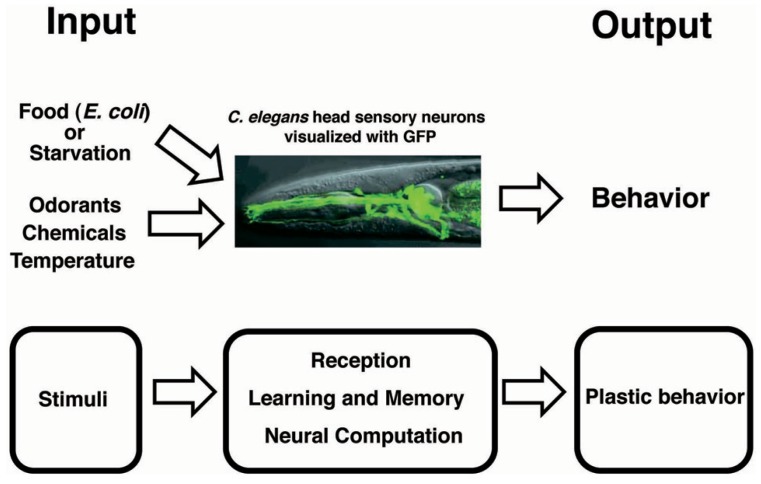
**Conceptual scheme for plastic behavior**.

*Caenorhabditis elegans* exhibits a rich repertoire of behaviors that are important for its survival and reproduction (**Figure 1**). Several studies have shown that *C. elegans* is not only capable of non-associative learning and short-term memory, but also capable of associative learning and long-term memory (Bargmann and Kaplan, 1998; Chalfie and Jorgensen, 1998; Rankin, 2002; Hobert, 2003; de Bono and Maricq, 2005; Giles et al., 2006; Giles and Rankin, 2009; Hart and Chao, 2010; Sasakura and Mori, 2013).

On the occasion of the Frontiers in Neural Circuits special issue on Japanese studies of neural circuits, we will describe the contributions of Japanese studies on *C. elegans* to the understanding of the neuronal bases of behavior. As mentioned above, molecules and neural signaling that underlie the plastic behavior of *C. elegans* are homologous to those found in mammals. Thus, the highly accurate genetic, neuronal, and behavioral studies on the *C. elegans* nervous system give great insights into our own nervous systems.

## REGULATION OF NEURAL CIRCUITS GOVERNING PLASTIC BEHAVIORS

### ENHANCEMENT OF ODOR RESPONSE ASSOCIATIVE LEARNING

Learning and memory are the fundamental neural processes in any animal, and the understanding of them is one of the most challenging fields in science (Kandel, 2001). *C. elegans* exhibits several behaviors that reflect two forms of learning: non-associative learning and associative learning (Bargmann and Kaplan, 1998; Chalfie and Jorgensen, 1998; Rankin, 2002; Hobert, 2003; de Bono and Maricq, 2005; Giles et al., 2006; Giles and Rankin, 2009; Hart and Chao, 2010; Sasakura and Mori, 2013). Non-associative learning is defined as a change in responses to a stimulus without association with a positive or negative reinforcement. In contrast, associative learning is defined as the process by which an association occurs between two different stimuli. *C. elegans* eats bacteria as a food source (Brenner, 1974; Avery and You, 2012). Food is the fundamental environmental cue for *C. elegans* and profoundly influences several of its behaviors. Dynamic changes in behaviors related to food are thought to be a behavioral strategy critical for acquiring food that may be scarce in nature. *C. elegans* associates food with several environmental stimuli, enabling worm researchers to study associative learning.

**FIGURE 2:**
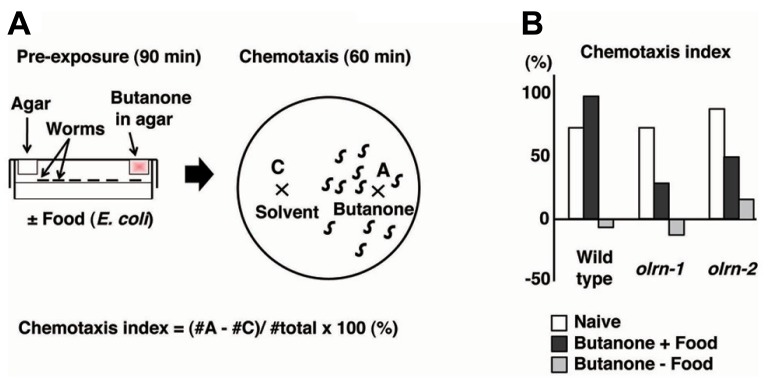
**The association of butanone and food to enhance chemotaxis to butanone (Torayama et al., 2007).** **(A)** Scheme for experiments. **(B)** Chemotaxis index of *olrn-1* and *olrn-2* mutants after butanone is associated with or without food.

Butanone and benzaldehyde are attractive odorants that are sensed by AWC olfactory neurons (Bargmann et al., 1993; Bargmann, 2006). Torayama et al. (2007) established a novel assay system whereby associative learning between an attractive odorant and food can be observed. They found that pre-exposure to butanone in the presence of food induces increased attraction toward butanone (**Figure 2**). Pre-exposure to butanone with food did not change the degree of chemotaxis to benzaldehyde, suggesting that this enhancement of chemotaxis was odorant-specific. The enhancement of chemotaxis to butanone did not require serotonin, which is responsible for food signaling, despite the essential role of food during the conditioning.

The forward genetic screen technique was used to isolate 10 mutants that showed limited butanone-enhanced chemotaxis. Of these, two mutants were further investigated and were found to show almost normal chemotaxis and adaptation to butanone, suggesting that the association between food and butanone is specifically impaired (**Figure 2B**). The first mutant, *olrn-1*, had a mutation in the gene encoding a novel transmembrane protein. Interestingly, *olrn-1* expression was strictly required in AWC neurons for the proper butanone enhancement. The left and right AWC neurons develop distinct sensory properties through an unusual stochastic lateral signaling interaction (Troemel et al., 1999; Wes and Bargmann, 2001). The best example of such regulation is the G-protein-coupled receptor STR-2, the expression of which is stochastically confined to either the left or right AWC neurons, resulting in the asymmetric expression of STR-2. The expression of STR-2 “on” cells is defined as AWC^ON^, and that of STR-2 “off” cells is defined as AWC^OFF^. AWC^ON^ and AWC^OFF^ express the different types of chemosensory receptors, thereby contributing to the sensation of different types of odors (Troemel et al., 1999; Wes and Bargmann, 2001). Notably, *olrn-1* mutants were defective in the asymmetry of AWC left and right cell fate and showed two AWC^OFF^ phenotypes, which suggests that the existence of AWC^ON^ is required for butanone enhancement (Bauer Huang et al., 2007; Torayama et al., 2007). Consistent with this hypothesis, another 2AWC^OFF^ mutant, *daf-11*, and the ablation of AWC^ON^ by a laser beam both caused the abnormal butanone enhancement.

Interestingly, 2AWC^ON^ mutants conversely showed defective butanone adaptation, in which animals pre-exposed to butanone showed an impaired chemotactic response to butanone, suggesting the importance of AWC^OFF^ for sensory adaptation. The asymmetric cell fate of AWC^ON^ and AWC^OFF^ is important not only for generating the diversity of sensation by expressing the different types of receptors, but also for generating the opposite types of plastic behaviors.

The second gene analyzed was *olrn-2*, which is identical to *bbs-8*, one of the Bardet–Biedl syndrome (BBS) genes (Torayama et al., 2007). BBS is a human genetic disorder that exhibits pleiotropic abnormalities, such as retinal dystrophy, polydactyly, renal malformation, and learning disabilities. Several BBS gene products are reported to be associated with ciliary biogenesis and functions (Ansley et al., 2003). The *C. elegans* genome contains at least eight homologs of human BBS genes designated as *bbs-1* to *-8*. They are all expressed exclusively in ciliated sensory neurons (Ansley et al., 2003). Indeed, *bbs-8/olrn-2* mutants showed structural defects in sensory cilia (Blacque et al., 2004). The expression pattern of STR-2 was normal in *bbs-8/olrn-2* mutants. Torayama et al. (2007) examined the dozens of *C. elegans* mutants defective in cilia structures and found that only *bbs* mutants showed abnormal butanone enhancement, although other cilia-defective mutants showed normal butanone enhancement, despite the fact that the chemotaxis to the butanone itself is impaired in these mutants. Torayama et al. (2007) revealed that butanone enhancement is specifically impaired in *bbs* genes, but the reason for this is unclear. Finding the reason for this may aid the understanding of BBS since BBS is known to cause learning disabilities.

### FORGETTING OF ODORANT MEMORY

Forgetting is the process of eliminating unnecessary or excessive information in the brain, enabling animals to obtain new information from their continuously changing environment. Despite the importance of forgetting, the neural and molecular bases of forgetting are almost unknown.

**FIGURE 3:**
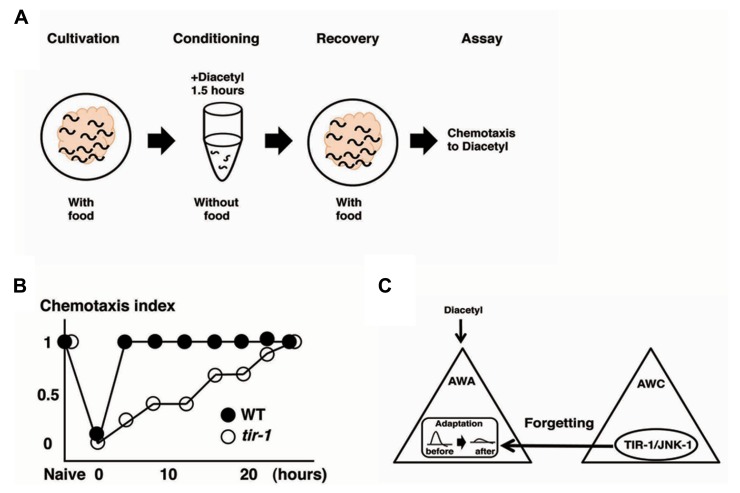
**The forgetting of adaptation to diacetyl is regulated through a TIR-1/JNK-1 pathway (Inoue et al. 2013).** **(A)** Scheme for experiments. **(B)** Retention curves of adaptation to diacetyl in wild type animals and *tir-1* mutants. **(C)** A model for the regulation of forgetting of olfactory adaptation to diacetyl. The TIR-1/JNK-1 pathway in AWC neurons regulates the forgetting process of adaptation in AWA neurons.

Olfactory adaptation is the plastic behavior in which animals pre-exposed to an odor in the absence of food show decreased response to the odor compared with naive animals. *C. elegans* shows attractive responses and adaptation to odorants sensed by two pairs of olfactory neurons, the AWA neurons and the AWC neurons (Colbert and Bargmann, 1995, Bargmann and Mori, 1997). Inoue et al. (2013) used the recovery from olfactory adaptation against AWA-sensed diacetyl as a behavioral paradigm for forgetting and found that the TIR-1/JNK-1 pathway regulates forgetting (**Figure 3**). *C. elegans* animals grown with food were exposed to diacetyl in foodless conditions for 1.5 h. Then, the animals were re-grown on food-rich plate for a certain period of time before a chemotaxis assay was performed (**Figure 3A**). Wild type animals pre-exposed to diacetyl for 1.5 h recovered normal chemotaxis to diacetyl within 4 h of exposure to food. In contrast, *tir-1* mutants isolated by forward genetics screen showed long-lasting adaptation: they gradually recovered the chemotaxis to diacetyl over about 25 h (**Figure 3B**). Calcium imaging revealed that the AWA response to diacetyl was suppressed during the adaptation period and recovered after 4 h in wild type animals, whereas the AWA response to diacetyl was still suppressed after 4 h in *tir-1* mutants. The sensory perception and the adaption to diacetyl were normal in *tir-1* mutants, suggesting that *tir-1* mutants are defective specifically in the process of forgetting. Notably, *tir-1* encodes a Toll/interleukin-1-resistance domain protein that is homologous to the mammalian adaptor protein SARM, which is known to be expressed in the brain.

In *C. elegans*, the TIR-1-mediated p38 mitogen-activated protein kinase (MAPK) pathway is known to regulate the asymmetric cell fate decision of AWC neurons. It also regulates the innate immune response. The UNC-43(CAMKII)-TIR-1-NSY-1(MAPKKK)-SEK-1 (MAPKK) pathway is required for the determination of AWC cell fate, whereas the TIR-1-NSY-1-SEK-1-PMK-1 (MAPK) pathway is required for the innate immune response (Troemel et al., 1999; Sagasti et al., 2001; Wes and Bargmann, 2001; Tanaka-Hino et al., 2002; Chuang and Bargmann, 2005; Shivers et al., 2009). Inoue et al. (2013) tested whether these molecular components were also involved in the forgetting process and found that *unc-43*, *nsy-1*, and *sek-1* mutants exhibited the similar forgetting defects to *tir-1* mutants, but *pmk-1* showed the normal phenotype. JNK-1 and PMK-1 are both MAPKs known to be phosphorylated by SEK-1 (MAPKK). The *jnk-1* mutants displayed forgetting defects, thus, the process of forgetting diacetyl is regulated through the TIR-1/JNK-1 pathway, which is partly overlapping but distinct from the pathways for AWC-asymmetric cell fate and the innate immune response. The TIR-1/JNK-1 pathway is critical for the forgetting process of AWC-sensed odor and of salt chemotaxis learning, suggesting the general role of the TIR-1/JNK-1 pathway in forgetting.

The neurons responsible for the process of forgetting diacetyl have been identified. Expression of *tir-1* cDNA into *tir-1* mutants under the AWA-specific promoter did not rescue the phenotype, but under the AWC-specific promoter rescued the defects. Expression of *sek-1* cDNA into *sek-1* mutants under the AWC-specific promoter also rescued the forgetting defects. Likewise, the expression of the dominant negative forms of *sek-1* and *jnk-1* only in the AWC neurons mimicked the forgetting-defective phenotype. These results suggest that the TIR-1/JNK-1 pathway in AWC neurons is sufficient and necessary for the forgetting process. Two pieces of evidence support the notion that the AWC cell itself plays an important role in the forgetting process of AWA neurons. First, *ceh-36* mutants, in which functional AWC neurons are undifferentiated because of developmental errors, exhibited forgetting defects (Lanjuin et al., 2003). Second, silencing of AWC neural activity by expressing the gain-of-function form of the UNC-103 potassium channel in AWC neurons induced the forgetting defects (Gruninger et al., 2008). The break of AWC-asymmetric cell fate mediated by the TIR-1/NSY-1 pathway is unrelated to the forgetting defects for the following reasons: *nsy-4* mutants, in which the asymmetry of AWC neurons is impaired, exhibited normal forgetting (Vanhoven et al., 2006); and *tir-1(gk264)* mutants, a special allele of the *tir-1* gene that retains AWC asymmetry, also exhibited forgetting defects. AWC neurons are critical for forgetting the adaptive process of AWA-sensed odor, and the TIR-1/JNK-1 pathway in the AWC neurons is essential for the forgetting process (**Figure 3C**).

The next question is how AWC neurons, apparently having no relation to diacetyl sensation, affect the forgetting events in AWA neurons. No neural connections have been reported between AWA and AWC neurons (White et al., 1986), so the information flow from AWC to AWA neurons cannot be explained by neural wiring. The secretion of some molecules by AWC neurons may play an important role for the regulation of forgetting events in AWA neurons. PKC-1, a novel protein kinase C-epsilon/eta is thought to regulate the synaptic release of neuropeptides and gain-of-function of PKC-1 is thought to activate the neuropeptide release at synapses (Okochi et al., 2005; Sieburth et al., 2007; Adachi et al., 2010). Expression of the gain-of-function form of *pkc-1* in AWC neurons rescued the forgetting defects in *tir-1* mutants, although it does not affect the chemotaxis and adaptation to diacetyl, suggesting that the TIR-1/JNK-1 pathway regulates neurosecretion from AWC neurons. Reciprocally, the expression of TetX (tetanus toxin light chain), an inhibitor of synaptic transmission in AWC neurons, caused wild type animals to exhibit the forgetting defects. Thus, AWC neurons may send a forgetting-accelerating signal to AWA neurons through secretion molecules. What induces AWC neurons to release the forgetting signal? The sensory perception of AWC neurons is not involved in forgetting, because *tax-4* mutants defective in AWC sensory signaling showed normal forgetting behavior (Komatsu et al., 1996). In contrast, whether animals were well fed or starved would be critical for AWC neurons to send forgetting signal to AWA neurons. Food signals could be captured in the synaptic region of the AWC axon to modulate TIR-1/JNK-1 signaling, thereby regulating the release of forgetting molecules. Consistent with this hypothesis, TIR-1 is localized at synapses (Chuang and Bargmann, 2005).

Inoue et al. (2013) clarified the existence of forgetting signals and showed that forgetting is not the passive decay of memory, but rather an active process. This study also revealed the hierarchical regulation of forgetting at molecular, cellular, and circuit levels. Further identifications of molecular components involved in forgetting, such as peptides and receptors, and the elucidation of neural regulation will help us understand the nature of forgetting.

### ENHANCEMENT OF REPULSIVE RESPONSE TO HARMFUL STIMULI THROUGH NON-ASSOCIATIVE LEARNING

In adaptation and habituation, which are classified as non-associative learning, sensory experiences weaken responses to stimuli. The opposite form of neural plasticity is also known, in which sensory responses are enhanced and animals overreact to previously experienced stimuli. The enhancement of sensory responses is critical for animals to escape from harmful environments. Well-known examples are the sensitization of mammalian peripheral pain sensations and the defensive responses in leeches and *Aplysia* (Hawkins et al., 1993; Sahley, 1995; Millan, 1999). Although addictive rather than defensive, drug addiction is another example of the enhancement of neural responses.

**FIGURE 4:**
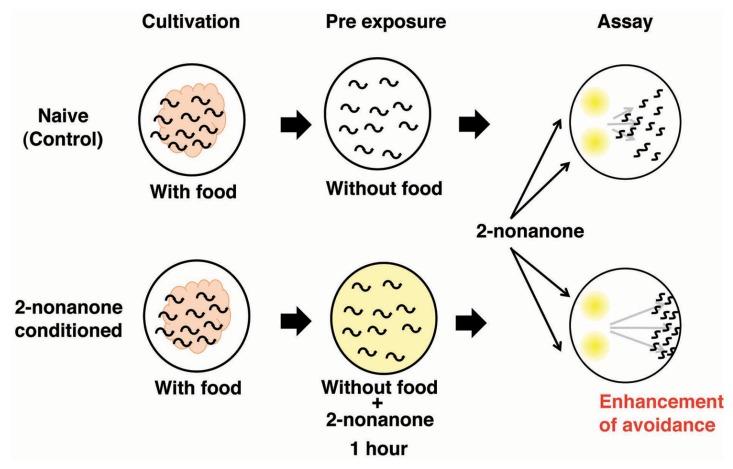
**The enhancement of avoidance behavior after the exposure to 2-nonanone (Kimura et al., 2004).** Scheme for experiments and the image for results.

An experimental system to explore the molecular and neural bases for sensory enhancement was established in *C. elegans* (Kimura et al., 2010). *C. elegans* shows avoidance behavior in response to the volatile odorant 2-nonanone, which is sensed mainly by AWB olfactory neurons (Troemel et al., 1997). Exposure to 2-nonanone for 1 h induced animals to move further away from the odor source than naive animals, and the enhancement of this escape response lasted at least 1 h (**Figure 4**). The enhancement of the touch response in *C. elegans* has also been reported, yet the molecular and neural bases for this are unknown (Rankin et al., 1990). The enhancement of 2-nonanone avoidance was not influenced by feeding state, hinting at the non-associative nature of this plasticity. Molecular genetic analysis revealed that dopamine signaling is involved in the enhancement of 2-nonanone avoidance. Notably, *dop-3* mutants lacking the D2-like dopamine receptor and dopamine biosynthesis-impaired mutants did not exhibit enhanced avoidance. In addition, the application of haloperidol, a D2-specific antagonist known as an antipsychotic drug, to wild type animals suppressed enhanced avoidance. The DOP-3 functioning neurons were investigated through cell-specific rescue experiments and a single pair of interneurons, known as RIC neurons, was identified as the site of dopamine action. The RIC neurons are known to be octopaminergic and it has been suggested that *dop-3* activity in the RIC neurons suppresses octopamine release (Suo et al., 2009). It has also been reported that RIC neurons form synaptic connections mainly with AVA neurons, which are involved in locomotion. Thus, dopamine signaling in RIC neurons may control the release of octopamine and/or the activity of AVA neurons. A video-based tracking system revealed the effect of 2-nonanone enhancement on escape behavior: the migration velocity remained constant, but the time taken to initiate escape behavior increased, as did the time taken to change the direction of movement. This technique, combined with the recently developed optogenetic system that enables researchers to control the neural activity of living animals during escape behavior, has shed light on the molecular and neural basis for sensory enhancement in *C. elegans* (Kawazoe et al., 2012).

### ASSOCIATIVE LEARNING BETWEEN ODORANT AND pH

*Caenorhabditis elegans* is able to sense environmental pH. Murayama et al. (2013) showed that a transmembrane receptor-type guanylyl cyclase GCY-14 may be the alkaline receptor in the ASEL gustatory neuron. Since food is a strong unconditional stimulus (US) in any conditioning paradigm, the behavioral protocol that avoids using food as US was established. Amano and Maruyama (2011) recently combined two defined chemical cues to analyze associative learning in *C. elegans*. They conditioned worms with 1-propanol as a conditioned stimulus (CS) and acidic pH as an US, and then conducted spaced training and massed training. Spaced training consists of repeated trials with an inter-trial interval (ITI), whereas massed training consists of repeated trials without an ITI. The memory after the spaced training was retained for 24 h, whereas the memory after the massed training lasted only 3 h. Consistent with the theory for touch response (Giles et al., 2006; Giles and Rankin, 2009), *C. elegans* likely processes both long- and short-term memories. Amano and Maruyama (2011) reported that the mutants defective in *nmr-1* encoding of an NMDA receptor subunit fail to form both long- and short-term memories, while mutations in *crh-1* encoding the CREB (cAMP-responsive element binding protein) transcriptional factor only affect long-term memory.

### NEURAL INTEGRATION OF TWO TYPES OF INFORMATION AND DECISION ON BEHAVIORAL CHOICE: A MODEL SYSTEM FOR DECISION-MAKING

**FIGURE 5:**
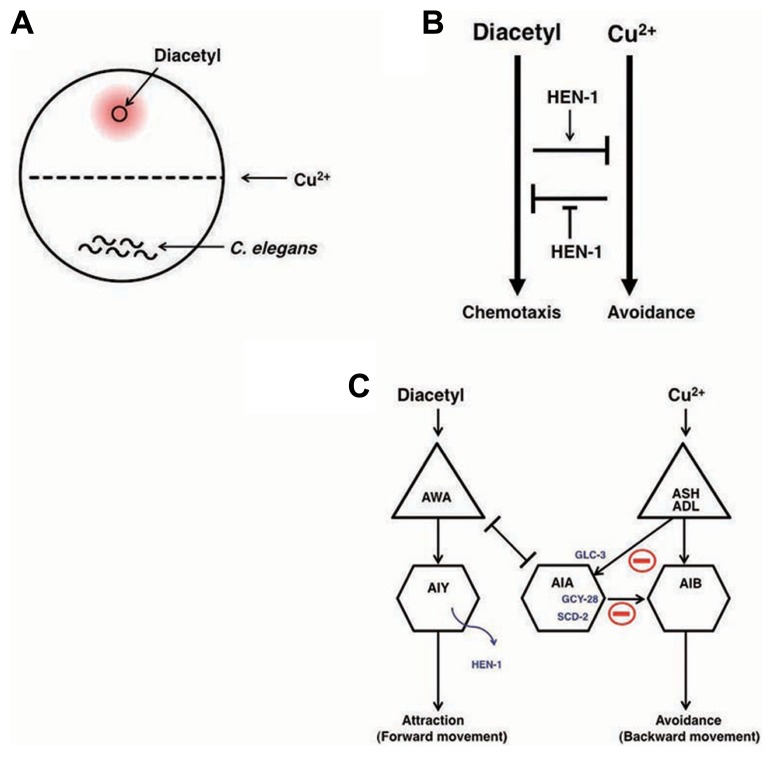
**Neural integration of two different types of information and decision on behavioral choice (Ishihara et al. 2002; Shinkai et al., 2011).** **(A)** The assay system of interaction assay. **(B)** The reciprocal inhibition model of the attractive signaling to diacetyl and the avoidance signaling to Cu^2^^+^. **(C)** A neural circuit model of the interaction of two sensory signals and decision-making. The activity of AIA interneurons is regulated by the balance between excitatory inputs from AWA through the gap junction and inhibitory inputs from ASH and ADL neurons through the glutamate-gated chloride channel GLC-3. Activated AIA interneurons would increase the relative strength of the signals for diacetyl by inhibiting the signals for Cu^2^^+^. GCY-28 and SCD-2 may enhance the inhibitory synaptic outputs from AIA neurons. HEN-1, a secretory protein released from AIY neurons, may act on the receptor SCD-2. The details of the mechanism of HEN-1 action are not yet fully understood.

Decision-making is the cognitive process by which animals select one action among several different choices. In order to study behavioral decision from two conflicting choices and the integration of two different sensory cues, an interaction assay system was developed (Ishihara et al., 2002). Diacetyl is an attractive odor sensed by AWA olfactory neurons and Cu^2^^+^ ion is aversive metal sensed by ASH and ADL sensory neurons (Bargmann et al., 1993; Sambongi et al., 2000). The attractive odor, diacetyl, is applied to one side of the assay plate, *C. elegans* animals are placed on the other side of the plate, and a Cu^2^^+^ barrier is established on the midline of the plate (**Figure 5A**). When *C. elegans* encounters the Cu^2^^+^ barrier during their migration toward diacetyl, the balance between concentrations of diacetyl and Cu^2^^+^ regulates the behavioral decision whether they go straight toward diacetyl or withdraw (**Figure 5B**).

The *hen-1* gene, which encodes a secretory protein with an LDL (low-density lipoprotein) receptor motif, regulates sensory integration and decision-making. In the interaction assay, Ishihara et al. (2002) found that *hen-1* mutants showed a weaker tendency to cross the Cu^2^^+^ barrier when approaching the attractive odorant diacetyl. Since *hen-1* mutants show a normal attractive response to diacetyl and aversion from the Cu^2^^+^ ion, the HEN-1 protein likely plays a role in decision-making in behavioral choice (**Figure 5B**). The cell non-autonomous function of the HEN-1 protein suggests that HEN-1 acts as a secreted molecule. Secretion of HEN-1 from AIY interneurons is crucial because *ttx-3* mutants (Hobert et al., 1997), which are defective in AIY cell fate, showed a similar phenotype to *hen-1* mutants.

The jeb protein in *Drosophila* is homologous to HEN-1 and has been reported to regulate the development of visceral mesodermal cells via the tyrosine kinase receptor Dalk (Weiss et al., 2001). SCD-2 is the sole *C. elegans* homolog of Dalk. The phenotype of *scd-2* mutants is almost the same as that of *hen-1* mutants, and *scd-2;hen-1* double mutants behave like both single mutants (Shinkai et al., 2011). These results suggest that *scd-2* and *hen-1* act via the same genetic pathway and support the idea that SCD-2 is a receptor of HEN-1. Forward genetic analysis identified the *gcy-28* gene as a key regulator for interaction behavior. GCY-28 is a membrane-bound guanylyl cyclase that produces cGMP (Tsunozaki et al., 2008; Shinkai et al., 2011). It was found that *gcy-28* was expressed broadly in neurons, however, the expression of *gcy-28.d*, one of the splicing isoforms, was expressed specifically in a pair of AIA interneurons. AIA interneurons were identified as the key neurons for the interaction behavior both through cell-specific rescue experiments and genetic cell-ablation experiments. The defects in *gcy-28* mutants were restored only when *gcy-28* cDNA was expressed in AIA neurons. Likewise, the genetic ablation of AIA neurons in wild type animals induced similar phenotypes to the *gcy-28* mutants. CNG-1 was identified as a cyclic nucleotide-gated channel (Cho et al., 2005) that functions downstream of GCY-28 in AIA neurons. Molecular genetic analysis suggested that the GCY-28/CNG-1 pathway is parallel to the HEN-1/SCD-2 pathway. The site of action of SCD-2 was also shown to be AIA neurons. Hence, both the GCY-28/CNG-1 and HEN-1/SCD-2 pathways are needed in AIA interneurons to act as integrators of conflicting sensory cues (**Figure 5C**).

The next question is how AIA neurons integrate sensory cues and make behavioral decisions. AWA olfactory neurons that are activated by diacetyl make gap junctions with AIA neurons, suggesting that AIA neurons can also be activated by diacetyl. However, ASH/ADL sensory neurons that are activated by Cu^2^^+^ form synapses with AIA neurons. ASH neurons are known to be glutamatergic, and the glutamate-gated chloride channel GLC-3 is known to be functional in AIA neurons (Chalasani et al., 2007). Interestingly, *glc-3* mutants crossed the Cu^2^^+^ barrier more frequently than did wild type animals and this phenotype was rescued by expressing *glc-3* cDNA in AIA neurons. These results suggest that ASH neurons activated by Cu^2^^+^ inhibit AIA neurons through the glutamate-gated chloride channel. The activity of AIA neurons is regulated in opposing fashion through AWA and ASH neurons: AWA neurons activate AIA neuron activity, whereas ASH neurons inhibit AIA neuron activity. The balanced regulation of AIA neuron activity may be important for behavioral decisions (**Figure 5C**). Although AIA neurons send synaptic outputs to many neurons, a major synaptic target of AIA neurons are AIB interneurons that are known to regulate a reversal of movement: removal of an odor sensed by AWC neurons activates AIB neurons to produce a reversal behavior, which reorients the animals to the odor source (de Bono and Maricq, 2005; Gray et al., 2005; Chalasani et al., 2007). In contrast, the application of an odor sensed by AWC neurons activates AIY neurons to induce forward movement that contributes to direct the animals straight to the odor source. Thus, AIA neurons and AIB interneurons have opposite effects on behaviors, hinting that AIA neurons activate inhibitory synapses on AIB neurons. Inhibitory connectivity from AIA to AIB neurons is also hinted by the laser ablation studies (Wakabayashi et al., 2004).

AIB interneurons receive synaptic inputs from ASH and ADL neurons, both of which sense aversive stimuli, including Cu^2^^+^. AIB may play a critical role in aversion behavior: ASH and ADL neurons activated by aversive stimuli convey the information to AIB neurons, and the activated AIB neurons induce reversal behavior, thereby enabling the animal to successfully escape from the aversive stimuli in the integration assay. Consequent activation of AIA interneurons that are connected via gap junctions to the activated AWA olfactory neurons may inhibit AIB neuronal activity, thereby preventing an aversive response to the Cu^2^^+^ barrier and accelerating migration toward diacetyl over the Cu^2^^+^ barrier. Consistent with this, activation of synaptic transmission of AIA neurons through AIA-specific expression of the gain-of-function form of *pkc-1* encoding nPKC-epsilon/eta (Okochi et al., 2005; Sieburth et al., 2007; Adachi et al., 2010) caused *gcy-28* mutants to cross over Cu^2^^+^ barrier. As discussed later, AIA neurons are important for associative learning (Tomioka et al., 2006), indicating that AIA neurons may function as controllers of neural plasticity (**Figures 5C and 7C**). Further analysis, such as clarification of the physiological properties of AIA neurons, identification and analysis of other molecules, and clarification of the relationship between these molecules will shed light on the decision-making process at the molecular and circuit levels.

### ALTERATION TO ODORANT PREFERENCE INFLUENCED BY POPULATION DENSITY OF ANIMALS

*Caenorhabditis elegans* strains isolated from natural environments all over the world are categorized based on behavioral properties into two group: social and solitary strains. Social strains show the aggregation of animals on the boundary of food where oxygen concentration is low. The standard laboratory strain of *C. elegans* is the Bristol type, which was isolated in England and exhibits the solitary phenotype (Brenner, 1974; de Bono and Bargmann, 1998; Gray et al., 2004; de Bono and Maricq, 2005). Social and solitary behaviors are regulated by FMRFamide-related neuropeptides and homologs of the neuropeptide Y receptor (de Bono and Bargmann, 1998; Rogers et al., 2003). It is important to consider the behavior of animals not only as individuals, but as populations.

As mentioned in the previous section, *C. elegans* stops approaching otherwise attractive odors and disperse from them after exposure to the odor in the absence of food (Colbert and Bargmann, 1995; Nuttley et al., 2002; Hirotsu and Iino, 2005). The density of animals has a large influence on this olfactory plasticity: animals grown in dense conditions exhibit a stronger tendency toward dispersion than the animals grown at a low density (Bowler and Benton, 2005). The interpretation of this phenomenon is that the association between the absence of food and an odorant helps animals to escape from foodless environments and motivates animals to explore new food sources. The high density of animals enhances the tendency toward dispersion and the tendency to explore new environments, because the animal judges that there is little hope of getting food due to the severe competition between individuals (Yamada et al., 2010).

**FIGURE 6:**
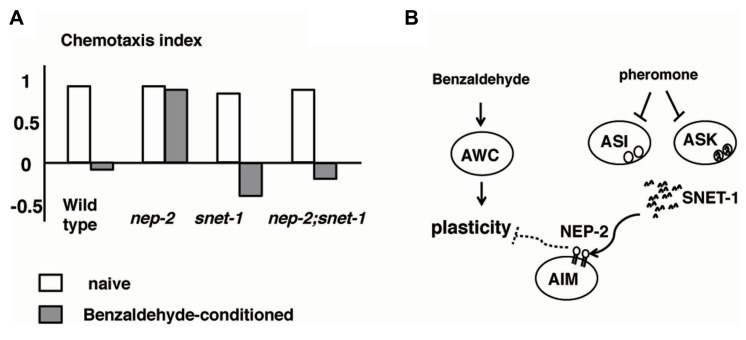
**Alteration to odorant preference influenced by population density of animals (Yamada et al., 2010).** **(A)** Olfactory plasticity of *nep-2*, *snet-1*, *nep-2:snet-1* mutants. The *nep-2* mutants exhibited defective olfactory plasticity. The abnormality olfactory plasticity of *nep-2* mutants is suppressed by the mutation in the *snet-1* gene. **(B)**. Proposed model of the alteration of olfactory plasticity induced by crude pheromone. SNET-1 is a peptide that inhibits olfactory plasticity. NEP-2 is an extracellular peptidase homologous to neprilysin. The environmental crude pheromone suppresses the expression of SNET-1, thereby facilitating the animals’ olfactory plasticity and ability to move away from the odor source. SNET-1 is accumulated in *nep-2* mutants causing the loss of olfactory plasticity and the migration to the odor source.

*Caenorhabditis elegans* recognizes the density of animals through a crude pheromone sensed by chemosensory neurons, including ASI neurons (Hu, 2007). When the crude pheromone was applied to *C. elegans*, the expression of SNET-1, a homolog of the *Aplysia* L11 peptide, was downregulated in ASI neurons. Yamada et al. (2010) found that SNET-1 is a signaling molecule that conveys the density of animals to the nervous system to regulate plastic behavior. The loss-of-function mutation of the *snet-1* gene mimicked high-density conditions and caused the enhancement of dispersion behavior after associative learning between the absence of food and benzaldehyde (**Figure 6**). On the other hand, the overexpression of *snet-1* weakened the tendency toward dispersion. The *nep-2* gene encoding the extracellular peptidase neprilysin was identified as the negative regulator that controls the activity of SNET-1. In *nep-2* mutants, the degradation of SNET-1 peptide is inhibited and the accumulated SNET-1 suppressed dispersion behavior. Taken together, the information on population density is transmitted through external pheromone and endogenous peptide signaling, thereby assuring behavioral plasticity that may be important for the survival of species (**Figure 6**; Yamada et al., 2010).

### ASSOCIATIVE LEARNING BETWEEN SALT AND FOOD

*Caenorhabditis elegans* displays chemotaxis to salt that is mediated mainly by ASE chemosensory neurons (Bargmann, 2006). A gene expression analysis of guanylyl cyclases suggested that ASEL and ASER neurons were different cells: *gcy-6* is expressed almost exclusively in ASEL, whereas *gcy-5* is expressed in ASER (Yu et al., 1997). The transcriptional control of left and right asymmetry has been examined in detail (Hobert et al., 2002; Ortiz et al., 2009). Laser ablation experiments have shown that left and right ASE neurons indeed sense different ions: ASEL senses Na^+^ and ASER senses Cl^-^ (Pierce-Shimomura et al., 2001). The physiological properties of ASE neurons were analyzed with electrophysiology and Ca^2^^+^ imaging. Whole-cell patch clumping of ASER neurons showed that ASER neurons are electrically isopotent, do not generate Na^+^ action potential, and are highly sensitivity to input currents over a wide voltage range (Goodman et al., 1998). Ca^2^^+^ imaging using a genetically encoded calcium indicator (GECI) showed that ASEL neurons responded to the addition of salt, whereas ASER neurons responded to the removal of salt (Suzuki et al., 2008).

**FIGURE 7:**
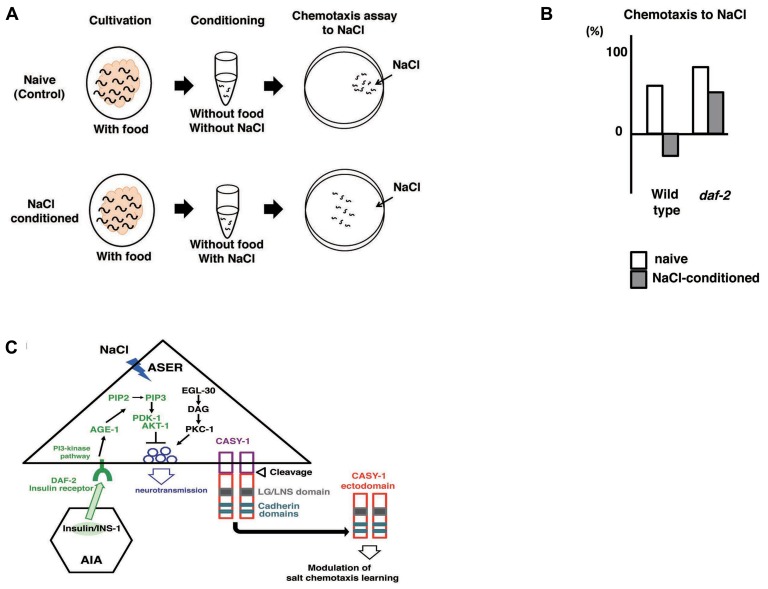
**Associative learning between salt and food (Saeki et al., 2001; Tomioka et al., 2006; Ikeda et al. (2008)).** **(A)** Scheme for experiments. **(B)** The *daf-2* mutants are defective in salt learning. **(C)** Proposed model for the regulation of salt learning. Insulin-like peptide INS-1 is secreted from AIA interneurons and the insulin-like signaling pathway is activated in the ASER neuron through the insulin receptor DAF-2. DAF-2 activates PI3 kinase AGE-1 that converts PIP2 to PIP3, which leads to activation of the downstream signal components Ser/Thr kinases PDK-1 and AKT-1. Then, the activation of this pathway negatively regulates neuronal activity of the ASER neuron, thereby generating plasticity in salt chemotaxis. EGL-30/Gq, DAG, and PKC-1/nPKC pathway in the ASER neuron may positively regulate the synaptic transmission antagonize to PI3 kinase pathway. Calsyntenins expressed in the ASER neuron is cleaved and the ectodomain is released. Released ectodomain acts on either the ASER neuron itself or others to modulate salt chemotaxis learning.

*Caenorhabditis elegans* subjected to prolonged exposure to salt under starvation conditions induced a dramatic reduction and negative chemotaxis to salt, suggesting that associative learning occurs between starvation and salt (termed “salt learning”; **Figure 7A**; Saeki et al., 2001; Hukema et al., 2006). Tomioka et al. (2006) showed that 10 min of starvation was enough to induce associative learning and that this learning lasted for approximately 1 h. Molecules involved in salt learning have been investigated. Hukema et al. (2006, 2008) showed that the G-protein, Ca^2^^+^, and cGMP pathways were involved in salt learning. Tomioka et al. (2006) revealed that the insulin-like signaling pathway played a critical role in salt learning (**Figures 7A, B**). The insulin-like signaling pathway is well known to regulate dauer formation (an arrested developmental variant) and aging in *C. elegans* (Hu, 2007). Mutants of *ins-1*, *daf-2*, *age-1*, *pdk-1*, and *akt-1*, which encode the homologs of insulin, insulin/IGF-1 receptor, PI3-kinase, phosphoinositide-dependent kinase, and Akt/PKB, respectively, showed severe defects in salt learning. INS-1 was secreted from AIA interneurons and localized to the synaptic regions of AIA neurons, which are connected to ASER neurons. Tomioka et al. (2006) showed that the DAF-2/PI3 kinase pathway in ASER neurons was required for salt learning. Their proposed model holds that INS-1-mediated feedback signaling acts on the salt receptor neuron ASER and activates the PI3 kinase pathway that may suppresses the synaptic releases from ASER neurons, thereby inhibiting the chemotaxis to NaCl (**Figure 7C**).

Suppression of synaptic release was supported by the finding that the activity of AIB neurons, one of the downstream interneurons of ASER neurons, is downregulated after salt learning (Oda et al., 2011). The molecular mechanism for synaptic transmission of ASER neurons has been partially elucidated. The expression of gain-of-function form of EGL-30/Gq, as well as gain-of-function form of PKC-1/nPKC in ASER neurons of wild type animals suppressed salt learning. In addition, PMA (phorbol myristate acetate), which is an analog of DAG and an activator of the PKC pathway, also suppressed salt learning. It is likely that the Gq/DAG/nPKC pathway promotes a subset of molecular activity that underlies the synaptic transmission of ASER neurons. The elucidation of the relationship between the Gq/DAG/nPKC pathway and the PI3 kinase pathway must be clarified in the future (Adachi et al., 2010).

Ikeda et al. (2008) screened mutants defective in salt learning and isolated *casy-1.* CASY-1 is a transmembrane protein carrying two tandem cadherin domains and an LG/LNS domain in the ectodomain. CASY-1 is an ortholog of calsyntenins (alcadeins) that is reported to be involved in episodic memory performance in humans (Ikeda et al., 2008; Hoerndli et al., 2009). The *casy-1* promoter::*gpf* was expressed broadly in neurons including ASE. The expression of *casy-1* solely in ASER neurons is sufficient to rescue the learning defects. In contrast, the defects were not rescued by expression in other neurons. These results indicate that CASY-1 plays a role in ASER neurons in salt learning. Ikeda et al. (2008) further showed that the ectodomain released through cleavage of CASY-1 is critical for salt learning (**Figure 7C**).

### CIRCUIT REGULATION OF ASSOCIATIVE LEARNING BETWEEN TEMPERATURE AND FOOD: A PROPOSED ANALOGY TO HUMAN BRAIN OPERATION

**FIGURE 8:**
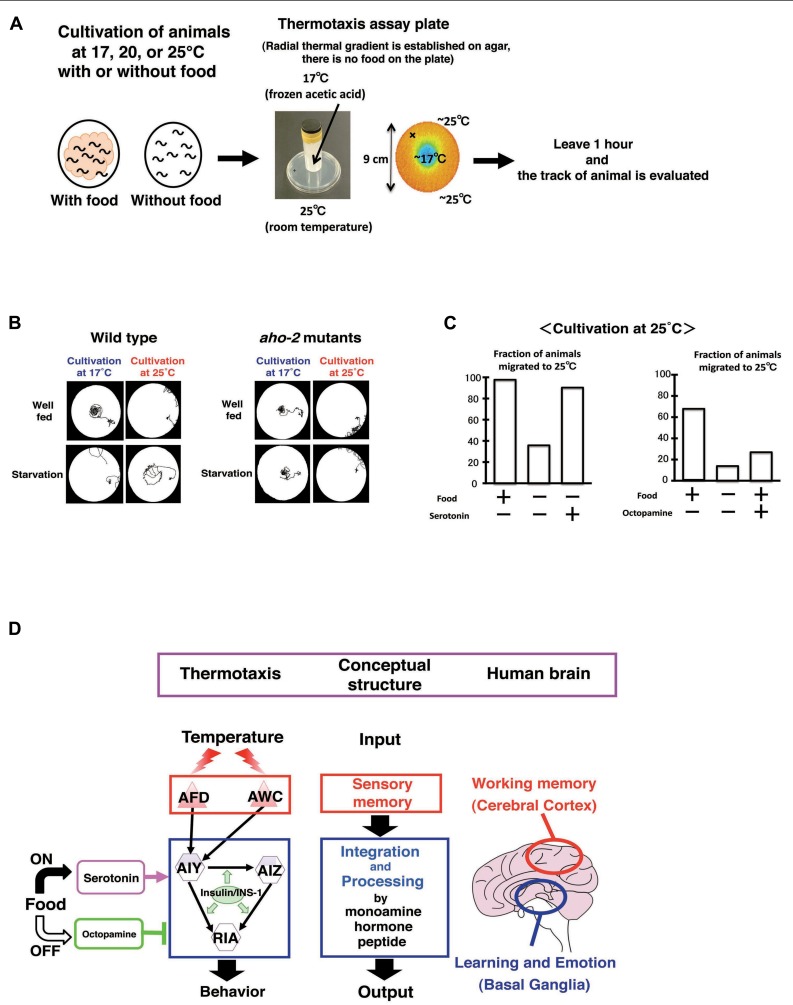
**Circuit regulation of associative learning between temperature and food (Hedgecock and Russell, 1975; Mori and Ohshima, 1995; Mohri et al. 2005; Kodama et al. 2006; Sasakura and Mori, 2013).** **(A)** Assay system and scheme for experiments. **(B)** Thermotaxis of wild type animals and *aho-2/ins-1* mutants. Wild type animals cultivated with food at 17 or 25°C migrate to the past growth temperature on a radial thermal gradient plate. In contrast, wild type animals cultivated without food (starvation conditions) diffuse and avoid the growth temperature area. The *aho-2/ins-1* mutants exhibited normal thermotaxis when grown with food, but exhibited abnormal thermotaxis when grown without food. Despite the starvation conditions, *aho-2/ins-1* mutants migrate to the past growth temperature. **(C)** Exogenous serotonin mimics the well-fed state and octopamine mimics the starvation state in thermotaxis. *C. elegans* grown at 25°C without food but with serotonin behaves like well-fed animals, whereas *C. elegans* grown with food but with octopamine behaves like starved animals. **(D)** Proposed analogy between the thermotaxis neural circuit in *C. elegans* and the human brain. Neural operation logic in *C. elegans* thermotaxis is analogous to that in the human brain. Stored thermal information in AFD neurons is transmitted to the thermotaxis core interneurons AIY, AIZ, and RIA. Thermal information and food state are integrated and processed in those interneurons by monoamines and insulin to generate output behavior. In the human brain, working memory is coded in the cerebral cortex, and the coded information is conveyed to the basal ganglia, where learning and emotion proceed with modulation through monoamines. We propose here a functional analogy between the simple neural circuit in *C. elegans* thermotaxis and the functionally layered structure of the human brain.

*Caenorhabditis elegans* associates past growth temperature with food. In its natural habitat, *C. elegans* likely adapts to the fluctuating temperatures in soil in order to stay near food sources. We can observe this behavior as thermotaxis in the laboratory (Hedgecock and Russell, 1975; Mori et al., 2007; Kimata et al., 2012; Sasakura and Mori, 2013). After animals were grown with food at a certain temperature ranging from 15 to 25°C and placed on an agar surface with a temperature gradient, they migrate toward the past growth temperature and move isothermally near that temperature (**Figures 8A–C**). Growth temperature-shift experiments indicated that the acquisition of a new temperature memory requires 2–4 h. Dynamic alternation of temperature preference is also induced by starvation. Growth without food at a certain temperature for several hours induces animals to disperse or avoid the past growth temperature (**Figures 8B, C**).

The simple neural circuit involved in thermotaxis is the perfect subject for the study of the functional connectome. In *C. elegans*, the environmental temperature is sensed by AFD and AWC sensory neurons, and then thermal signals are transmitted to the downstream interneurons AIY, AIZ, and RIA. The AIY-mediated neural pathway is responsible for thermophilic movement, whereas the AIZ-mediated neural pathway is responsible for cryophilic movement. The counterbalanced regulation of the activities of AIY and AIZ neurons is thought to be essential for thermotaxis. The RIA interneurons integrate signals from both upstream interneurons AIY and AIZ neurons. Consequently, the outcome of neural computations in the core thermotaxis circuit regulates the body wall muscles, thereby controlling the worm’s ultimate behavior (**Figure 8D**).

In addition to their thermosensory function, AFD neurons also serve as a temperature memory device. The memory function of AFD neurons was revealed by Ca^2^^+^ imaging, where the response of AFD neurons to warming corresponded to their past growth temperature (Kimura et al., 2004; Clark et al., 2006; Mori et al., 2007; Kimata et al., 2012; Sasakura and Mori, 2013). The CREB is a transcriptional factor that regulates neural plasticity from invertebrates to mammals. Nishida et al. (2011) showed that the mutants in *crh-1* gene encoding a *C. elegans* homolog of CREB showed abnormal thermotaxis and the expression of *crh-1* cDNA only in AFD neurons almost completely reversed the defects. CREB function in AFD may be required for temperature memory or the presynaptic plasticity.

Kodama et al. (2006) showed by Ca^2^^+^ imaging that food or starvation signals did not affect AFD neuronal response to temperature. These results imply that food or starvation signals influence the neural activity of interneurons in the circuit, thereby generating plastic behaviors. Molecular genetic analysis has identified the several molecules critical for the associative learning between food and temperature. The *aho-2* mutation, isolated in a forward genetic screen, was identical to the *ins-1* mutation, suggesting the general role of insulin in learning (Kodama et al., 2006; Tomioka et al., 2006). INS-1 acts in a cell non-autonomous fashion in interneurons required for thermotaxis. INS-1 antagonizes the DAF-2/insulin receptor and the AGE-1/PI3 kinase pathway. The behavioral defects of *age-1* mutants were rescued by expressing the *age-1* gene in any of three interneurons, AIY, AIZ, and RIA, all of which are component interneurons in the thermotaxis circuit. Thus, INS-1 acts on the interneurons to change the dynamics of the neural circuit. These results are in contrast to salt learning, in which INS-1 acts on the salt-sensing neuron ASER (Tomioka et al., 2006).

An ortholog of the human calcineurin alpha subunit TAX-6 is required in two pairs of interneurons, AIZ and RIA, for the associative learning between food and temperature (Kuhara and Mori, 2006). A novel type of hydrolase AHO-3 is important for this learning (Nishio et al., 2012). The expression of FAM108B1, which is a human homolog of AHO-3 and strongly expressed in the human brain, restored the defects of *aho-3* mutants. ABHD6, another protein related to AHO-3, was reported to be involved in endocannabinoid signaling in mouse neural culture cells (Marrs et al., 2010). Hence, endocannabinoid signaling could be involved in the associative learning between food and temperature.

Exogenous serotonin mimics the presence of food, whereas exogenous octopamine mimics the absence of food in many aspects of *C. elegans* behavior, such as locomotion, pharyngeal pumping, and egg laying (Horvitz et al., 1982; Sawin et al., 2000; Chase and Koelle, 2007). Mohri et al. (2005) found that the application of serotonin to food-deprived animals induces thermotaxis to a memorized temperature, while the application of octopamine to well-fed animals suppresses thermotaxis to a memorized temperature (**Figure 8C**). These results suggest that the balanced regulation through these two monoamines in interneurons is a key process for thermotactic plasticity. It is intriguing to ask how the antagonistic behavioral regulation through serotonin and octopamine is related to the antagonistic neural regulation between AIY and AIZ neurons (Mori and Ohshima, 1995). It is also important to uncover the relationship between insulin signaling and monoamine signaling. A recent unexpected finding is that temperature information sensed by non-neuronal tissues such as intestines and body wall muscles through the heat shock transcriptional factor-1 (HSF-1) feeds back to the core thermosensory circuit and regulates the activity of AFD neurons via estrogen signaling (Sugi et al., 2011).

We propose that the neural circuit for thermotactic plasticity is amazingly similar to two functional parts of the human brain: the cerebral cortex and basal ganglia (**Figure 8D**; Sasakura and Mori, 2013). The cerebral cortex encodes the working memory that is required for the temporal storage of information. The basal ganglia play an important role in learning, emotion, and motivation. In the neural circuits for themotaxis, temperature information stored in the sensory neurons AFD and probably AWC, is transmitted to the interneurons, AIY, AIZ, and RIA, where the temperature information is associated with feeding and/or starvation signals to generate associative learning. Thus, the sensory neurons AFD and probably AWC, working as a memory storage device, are equivalent to the cerebral cortex, while the three interneurons AIY, AIZ, and RIA, which play a part in learning and likely receive neuromodulatory monoamines according to feeding states, are equivalent to the basal ganglia (**Figure 8D**).

There is mounting evidence to support the notion that the *C. elegans* nervous system has many physiological similarities to the human brain. Serotonin and dopamine signaling regulates the motivational behavior in locomotion in *C. elegans* (Sawin et al., 2000). The serotonin reuptake inhibitor fluoxetine (Prozac) is able to change locomotion behavior in *C. elegans* (Choy and Thomas, 1999; Ranganathan et al., 2000, 2001). Catecholamine modulates the sensory stimulus from environmental food to determine whether *C. elegans* remains in the vicinity of food (Bendesky et al., 2011). Vasopressin/oxytocin signaling, which regulates water balance, reproduction, and social behavior in mammals, is critical for gustatory associative learning and reproductive behaviors (Beets et al., 2012; Garrison et al., 2012). Sleep-like behavior and EGF signaling, which are important for the day–night cycle behavior in mice, is also critical for *C. elegans* rest-taking behavior (Van Buskirk and Sternberg, 2007; Raizen et al., 2008).

The *C. elegans* genome contains nearly the same suit of neuronal genes as humans: transcriptional factors, components of synapses, gap junctions, neurotransmitters, neuromodulators, receptors, ion channels, and so on (Bargmann, 1998; C. elegans Sequencing Consortium, 1998; Hobert, 2005; Jorgensen, 2005; Brockie and Maricq, 2006; Chase and Koelle, 2007; Rand, 2007; Li and Kim, 2008). Their genome-level similarity suggests that the neural bases underlying behavior are indeed similar. In addition, recent work on connectivity patterns in the male *C. elegans* nervous system tells us that principles of the *C. elegans* neural network are also similar to those of the human brain (Jarrell et al., 2012). Given that neural plasticity is necessary for survival of the animal from an early stage of evolution, the principles of neural function that underlie behaviors may be conserved between *C. elegans* and humans.

## DISSECTION OF INFORMATION FLOW AND PROCESSING IN NEURAL CIRCUITS THROUGH ADVANCED TECHNOLOGY: IMAGING, OPTOGENETICS, AND TRACKING SYSTEMS

### MONITORING NEURAL ACTIVITIES

Monitoring intercellular activity is important for understanding the mechanism of neural circuits. The first GECI, called cameleon, was invented in 1997 by Miyawaki et al. (1997). Deployment of GECIs in biological sciences, particularly in neurosciences, has accelerated the development of various GECIs (Nakai et al., 2001; Tian et al., 2009; Zhao et al., 2011; Akerboom et al., 2012; Chen et al., 2013). Attempts to develop new and modified GECIs have greatly promoted studies of neural circuits as well. Calcium imaging techniques are powerful tools, because calcium signals transduce a variety of information in tissues and organelles. Other monitoring techniques also have the potential to enable the observation of different types of intercellular events. Tomida et al. (2012) monitored activity of MAPK in the salt-sensing sensory neuron ASER in living worms. Using a Förster resonance energy transfer (FRET)-based probe, they showed that MAPK activity corresponded to salt stimulus depending on its given patterns of repetitive salt pulses. Comparison between the time course of MAPK and Ca^2^^+^ activities showed that MAPK activity is related to the non-linear response of Ca^2^^+^ to the given salt patterns. These results shed light on the modulation mechanisms of signal transduction inside cells that respond to the environmental signals.

For the purpose of monitoring neural activity, neuroscientists require good voltage-sensitive fluorescence sensors. Although voltage-sensitive dyes have certain advantages such as to allow direct monitoring of the signals of neural activity, recording both synaptic input and action potential output, and comparing the acquired data with electrophysiological results, their low signal-to-noise ratio limits their practical use. *In vivo* electrical recording using genetically encoded voltage indicators (GEVI) is a particularly challenging problem. Using the voltage-sensitive fluorescent protein VSFP 2.42 (Akemann et al., 2010), activity of AIY interneuron was explored by Shidara et al. (2013). They reported different dynamics of the neurite and the soma in response to given odorant stimuli. Since some interneurons, such as RIA and AIY, show compartmental activity of calcium dynamics (Clark et al., 2006; Hendricks et al., 2012), different types of imaging probes are necessary to address whether different locations in neurons have different functions.

### BEHAVIORAL ANALYSIS WITH AUTOMATED TRACKING SYSTEMS

Behavior is the eventual output of neural circuits. Behavioral analysis therefore clarifies the meanings and functions of each neural mechanism. To perform behavioral analysis, automated data acquisition and computational methods are useful to increase throughput of tedious experiments and to exclude the subjectivity of experimenters. In fact, computational methods are helpful to categorize each observed behavioral component based on rigid criteria by describing the criteria in computer languages. Long-term behavioral analysis has the merit of using a computational approach since computers can perform a repetitive analysis an almost infinite number of times. Statistical analysis based on automatically acquired abundant data may yield a subtle difference in the properties that are usually hidden in the noise of observed measurements. For the analysis of *C. elegans* behavior, several tracking systems have been developed in different labs (Husson et al., 2013a).

Hoshi and Shingai (2006) developed a tracking system for the automated analysis of the locomotion of *C. elegans*. Their system identifies the locomotory state (forward movement, backward movement, rest, and curl) of worms on agar plates using several hours of data. The heads and tails of tracked worm are also identified from acquired images using their algorithm (Hoshi and Shingai, 2006). Using this system, Wakabayashi et al. (2004) identified distinct behavioral states during forward locomotion. The addition of the laser ablation technique further allowed the quantification of the relationship between the behavioral state and related neurons (Wakabayashi et al., 2004). Visualizing locomotory behavior with computational measurements itself is also useful for discriminating behavioral phenotypes. Miyara et al. (2011) analyzed the role of a novel protein called macoilin with a tracking system by visualizing locomotory undulation of *C. elegans*. Similar methods have been developed by several labs and this approach is becoming popular to describe the locomotory undulation of *C. elegans* (Korta et al., 2007; Pierce-Shimomura et al., 2008; Stephens et al., 2008; Fang-Yen et al., 2010).

### OPTOGENETICS OF NEURAL CIRCUITS

Optogenetic techniques enhance the validity of tracking systems by adding non-invasive operation for neural activities using photo-activation of ion channels or pumps on the target neurons during long-term observation of freely moving animals. The combination of neuronal perturbation and behavioral monitoring is a powerful approach for understanding the mechanisms of behavioral control by neural circuits. A pioneering work that combined a tracking system and optogenetics was done in order to analyze thermotaxic behavior (Kuhara et al., 2011). A yellow light stimulus enabled to weaken the activity of the thermosensory neuron AFD with a transgenic line expressing halorhodopsin coding a light-activated chloride pump. Together with a tracking technique, mutant analysis, and calcium imaging for AFD neurons and the downstream interneurons AIY, Kuhara et al. (2011) showed that the AFD–AIY circuit could regulate the activity of AIY neurons in both excitatory and inhibitory directions, which accounts for thermophilic and cryophilic movements, respectively.

While the laser ablation technique completely disrupts activity of the target neuron, optogenetic methods perturb the target neural circuits quantitatively in a specific time window. Long-term observation of such quantitatively perturbed target animals would be beneficial for uncovering the detailed mechanisms of neural circuits. Studies with powerful new light-driven proton-pumps such as Arch (Chow et al., 2010; Okazaki et al., 2012; Husson et al., 2013b) and as ArchT (Okazaki and Takagi, 2013) in *C. elegans* research has enabled very effective and long-term silencing of neurons, under continuous illumination for up to 1 min, at a minimum. When an Arch mutant (M128A/S151A/A226T), designed to cause a blue shift in the action spectrum, is expressed in *C. elegans* neurons, locomotory arrest can be elicited by blue light, proving that *C. elegans* locomotion can serve as a convenient assay for *in vivo* evaluation of new optogenetic tools (Sudo et al., 2013).

### MANIPULATION OF SPATIOTEMPORAL GENE EXPRESSION

A previous report indicated that available cell-specific promoters enable unique expression of transgenes in only 12% of neuronal groups (Chelur and Chalfie, 2007). Since some promoters only drive gene expression relatively weakly, the number of promoters with practical utility for driving transgene expression in unique neuronal types is further limited. Several intersectional approaches utilize a pair of promoters with overlapping specificities to express a target gene in a single neuronal group. Split GFP and split apoptotic factor can function following the intersectional gene expression of each partner fragment, and an FLP-out strategy using *in vivo* recombination systems such as Flp-FRT or Cre-loxP enables expression of a given protein with higher cell-type specificity. However, it is still impossible to express a particular gene in a single targeted neuron, or a combination of multiple targeted neurons.

Inducible promoters are also frequently used for temporal regulation of transgene expression in model organisms. Given the targeting of external stimuli to a defined local region, the use of inducible promoters can enable spatially targeted induction of genes, even at single cell spatial resolution. The use of heat-activated heat shock promoters has been explored, and attempts have been made to deliver heat locally. Lasers with high spatial resolution can target small areas in tissue for local heating, a promising approach for activating transgenes under the control of heat shock promoters. The heat shock response, which is aided by the heat shock promoter, leads to activation of transcription. Since the heat shock response is a physiological defense mechanism inherent in almost all cell types in most organisms, such methods would have broad applicability in biological studies.

Irradiation with a Coumarin 440 dye laser beam under microscopic control is a common technique for cell ablation in *C. elegans* and other organisms. Assuming that heat generated by the laser is the main cause of cell death, attempts have been made to induce the heat shock response in targeted cells without causing cell death, by reducing the laser power level. Pioneering researchers have reported successful laser-mediated heat shock induction in single targeted cells in *C. elegans*, *Drosophila* and zebrafish (Stringham and Candido, 1993; Halfon et al., 1997; Halloran et al., 2000). However, there have been few follow-up studies using this method because it was found to have two major drawbacks: low efficiency of gene induction, and cell damage caused by irradiation (Harris et al., 1996; Kamei et al., 2009). Thus, it appears that the Coumarin 440 dye laser is not a good choice for heating cells.

### THE IR-LEGO SYSTEM

The IR-LEGO (infrared laser-evoked gene operator) is a novel recently developed system that uses an infrared (IR) laser radiating at 1480 nm, a wavelength at which the absorption coefficient of water is about 10^5^ times higher than that at 440 nm (Kamei et al., 2009; Suzuki et al., 2013). The much higher absorption rate enables efficient heating of water in specimens so that the heat shock response can be effectively induced using relatively low input power, which in turn helps avoid photochemical damage during irradiation. The IR-LEGO system was first applied to *C. elegans*, and the induced gene expression was successfully demonstrated in targeted single cells for multiple cell types, including neurons (Kamei et al., 2009). By choosing an appropriate laser power level, gene expression can be induced at a frequency of around 50% using short irradiation periods (less than 1 s), a much shorter duration than those (75 s to 10 min) reported in gene induction experiments using the Coumarin 440 dye laser. Following irradiation, targeted cells showed no apparent damage; they expressed marker genes, executed cell divisions, and eventually completed differentiation normally. Induction of wild type gene expression in single mutant cells has also confirmed that phenotypic defects such as cell migration or fate determination can be successfully rescued. The IR-LEGO system’s induction efficiency, which is sufficient for practical use, and its lack of harmful effects indicate that the problems inherent in earlier laser-mediated gene induction methods have been overcome. The IR-LEGO system can be applied to many transparent organisms, and can be equipped for transgenic technology. To date, it has been used on fish (*Oryzias latipes* and *Danio rerio*) and on a higher plant (*Arabidopsis thaliana*; Deguchi et al., 2009; Kimura et al., 2013). In fish, cells at a depth of 150 μm were induced to express a transgene using the IR-LEGO system.

An *in vitro* study using a polyacrylamide gel tissue model has suggested that temperatures at the irradiation focus change very quickly in response to IR irradiation, and that the area with temperature shifts exceeding 20°C was essentially confined to a vertically extended ellipsoidal area 7 μm along the *x*–*y* axes (Kamei et al., 2009). Although this size is sufficiently small to heat individual cells in most organisms, the original continuous irradiation procedure of the IR-LEGO system sometimes induces gene expression in multiple cells in *C. elegans* when the target lies in a densely packed cell cluster such as a ganglion. This may reflect the more limited dissipation of heat in living organisms compared with that in the *in vitro* model. In order to achieve efficient gene induction in single neurons in ganglion by using the IR-LEGO system, a promising approach is to use pulsed irradiation that minimizes the heating of areas surrounding the focus area by facilitating heat dissipation during interpulse periods. When combined with an FLP-out strategy, the IR-LEGO system can be used for inducing sustained gene expression in single targeted neurons, broadening its potential for future applications.

## THEORETICAL APPROACHES

Theoretical approaches promote the understanding of the mechanisms of neural circuits by providing an integrated view of different experiments or bringing new insights into the analyses of the complex data. With this in mind, computational simulations can validate hypothesized rules, especially when several rules are combined.

As described above, *C. elegans* exhibits food and temperature associative learning behavior called thermotaxis, where animals grown at a constant temperature with food migrate to the previous cultivation temperature on a thermal gradient. For the analysis of thermotaxis, several theoretical studies have attempted to explain different results of thermotaxis behavioral assays in different conditions.

Although the original study on this topic reported both cryophilic and thermophilic migration, where the animals migrate toward colder and warmer regions, respectively, some reports have shown the absence of thermophilic migration under certain experimental conditions (Hedgecock and Russell, 1975; Ryu and Samuel, 2002; Yamada and Ohshima, 2003; Kimata et al., 2012).

Based on a biased random walk model for *C. elegans* searching behavior, Matsuoka et al. (2008) examined the inconsistency between the results of population and individual thermotaxis assays used in the different reports. The Monte Carlo approach, which involves random sampling from a numerical model, showed the simulated population dynamics of *C. elegans* thermotaxis based on the migration rules for individual worms. In spite of the lack of thermophilic rules above growth temperatures for hypothetical individual worms, the simulated population dynamics showed a thermophilic tendency. Thus, the simulation results were consistent with the different results in various experimental conditions.

Nakazato and Mochizuki (2009) took another approach to deal with the problems that include inconsistency in thermotaxis assays in the different reports. They constructed a mathematical model based on the differential equations for the population dynamics of *C. elegans* migration during thermotaxis. With hypothetical parameter sets based on the previously published experimental data, they examined the results of thermotaxis assays in the different reports. In spite of the inconsistent results of previous theoretical reports, the proposed model generates consistent results for each experiment with the same parameter set.

In contrast to the above behavioral modeling studies, a detailed computational neural model for dynamic body regulation elucidates different aspects of the modeling study. Suzuki et al. (2005) constructed a computational model for locomotory regulation of *C. elegans* based on the known anatomical neural wiring map. Even with the complete anatomical neural wiring map of 302 neurons of *C. elegans*, orchestration of each motor neuron for locomotory movements, including the location of the central pattern generator, is still unclear. Simulation approaches allow the reconstruction of the dynamic behavior of a system combining the knowledge of the components of the system. Describing the dynamics of the whole system that consists of multiple components is helpful for understanding the orchestration inside the system; once a model is constructed, a survey of the effects of each component is feasible. Suzuki et al. (2005) modeled the locomotory movement of *C. elegans* with a motor neuronal network and regulated body using a multi-joint rigid link model. Then they reconstructed the movements of wild type animals and *unc-25* mutants that lack GABA inhibitory signals in motor neurons. Such a reconstruction approach of the dynamic system is helpful for understanding the relationship between each component and the whole system.

Another theoretical approach involves observing quantitatively measured experimental data in combination with computational simulations. Ohkubo et al. (2010) analyzed trajectory data of worms during NaCl chemotaxis. Using cumulative distributions of curving rates in a log-log scale, comparison with the Gaussian distribution showed the long-tail behavior of curving rate of the worm trajectories (Ohkubo et al., 2010). The long-tail property of the curving rate implies the multiplicative noise of neural outputs, which inherit noise term by multiplication, although the long-tail behavior is not restricted to the specificity of the multiplicative noise. They constructed mathematical models based on the random walk model, and the simulation results showed the robustness of the long-tail property in several conditions. As shown by Ohkubo et al. (2010), the analysis with the theoretical standpoint would help extract the essence of the system’s properties.

Particularly for the complex objects like neural circuits, computational results do not assure the validity of hypothesized model, since biological systems usually include many unknowns. However, theoretical approaches and computational simulations provide logically descriptive tools for dynamic systems to develop working hypotheses. Mutual feedback between experimental and theoretical studies will advance the understanding of complex phenomena such as behavioral regulation by neural circuits.

## SUMMARY AND PERSPECTIVE

As we have reviewed above, studies using *C. elegans* have carried neural circuit studies forward. They include studies on the complex functions of nervous systems such as learning and memory. The understanding of the design of behavioral assay captures the essence of complex neural functions; the simple neural circuit of *C. elegans* is well suited for the detailed investigation for the target mechanism. Development and application of novel technologies accelerate the progress of research and also shed light on the hidden aspects of neural mechanisms. The Japanese *C. elegans* research community contributes to advance the neural circuit studies by providing unique behavioral assay frameworks and technological developments. Behavioral and neural circuit studies of *C. elegans* have furnished molecular and mechanical insights into neural operation to the understanding of the human brain. This pioneering role of *C. elegans* for neuroscience is expected to continue to be important for human brain studies. “The Brain Activity Map Project,” declared by Barack Obama, President of United States, in April 2013, is a large-scale, 10-year continuous project that finally aims to map the activity of every neuron functional connection in the human brain (Alivisatos et al., 2012, 2013). This large-scale project is equivalent to the “Human Genome Project” in which *C. elegans* genome studies played a pivotal role as a pilot case. The already identified complete connectome (302 neurons and approximately 7,000 connections) and full recording of all neuronal activity in the near future will be an important boost for the brain activity map project. Given this situation, *C. elegans* neuroscience is becoming more valuable than ever.

## Conflict of Interest Statement

The authors declare that the research was conducted in the absence of any commercial or financial relationships that could be construed as a potential conflict of interest.
